# Octabetaines: a DFT Study of Unexplored Eight-Membered 10π Heterocycles

**DOI:** 10.1021/acsomega.4c10544

**Published:** 2025-01-28

**Authors:** Christopher A. Ramsden, Wojciech P. Oziminski

**Affiliations:** a Lennard-Jones Laboratories, School of Chemical and Physical Sciences, Keele University, Staffordshire ST5 5BG, U.K.; b Department of Organic and Physical Chemistry, Faculty of Pharmacy, Medical University of Warsaw, 1 Banacha Street,Warsaw 02-097, Poland

## Abstract

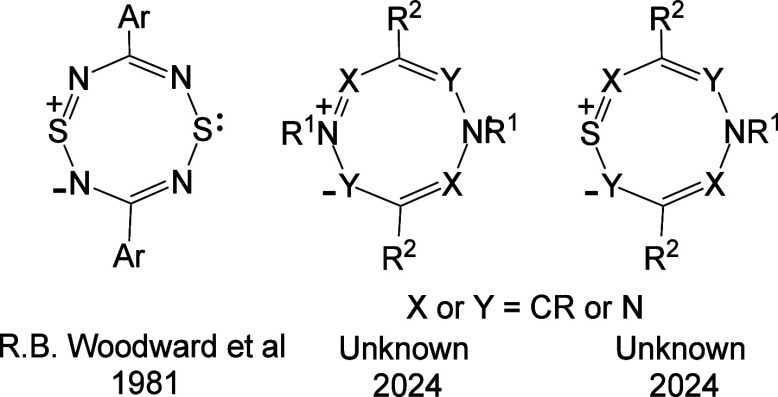

Eight-membered 10π heterocyclic mesomeric betaines (HMBs) are unusual in that they are associated with two 1,3-dipolar fragments in mutual conjugation; they are conjugated HMBs belonging to Class 1A. Apart from the characterization of five derivatives of the 1,5-dithia-2,4,6,8-tetrazocine ring in the 1980s, this large family of “aromatic” heterocycles has received no attention. The density functional theory (DFT) study reported here investigates the structure, aromaticity, and bonding of representative examples. The parent structures and aza derivatives are planar, and bond lengths are in agreement with a reported crystal structure. Calculated HOMA aromaticity index values and aromatic stabilization energy (ASE) are consistent with high classical aromaticity. Their magnetic aromaticity, measured by the NICS(1)_*zz*_ index and π electron current density maps, is also high. The introduction of electron-donating ring substituents (Me, OH, and NH_2_) results in distortion from planarity. The energy values of the frontier orbitals, vertical ionization potentials (VIPs), and vertical electron affinities (VEAs) are reported. Their significant variation with position of aza substitution is rationalized by a perturbation model based on the frontier orbitals of the cyclooctatetraene dianion.

## 1 Introduction

The structures **1a** (*X* or *Y* = NR, S) represent the parent structures of a large unexplored family of eight-membered 10π heterocycles. To provide further insight into the structures and properties of this class of HMBs, we describe a DFT study of betaines **1a** (*X* or *Y* = NH, S) and their aza derivatives.
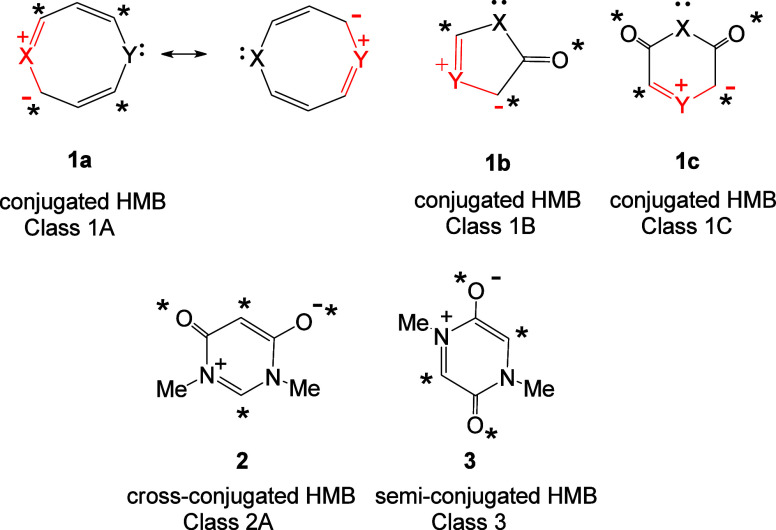


Three discrete types of HMB have been identified and named as (i) conjugated (Class 1), (ii) cross-conjugated (Class 2), and (iii) semiconjugated (Class 3) HMBs.^1−3^ Subclasses of each class (denoted by A, B or C) occur depending on the positions of connection (starred or unstarred) of the 2π heteroatoms (i.e., *X* and *Y*) to the odd alternant π fragments of the ring. The most studied class is conjugated HMBs (Class 1), which are associated with 1,3-dipolar fragments within the ring. Three subclasses of conjugated HMBs occur (Classes 1A–C). The betaines **1a** belong to Class 1A, and their structures are characterized by two 1,3-dipolar fragments in mutual resonance. Class 1B conjugated HMBs include type A mesoionic rings **1b**.^4^ Class 1C HMBs are represented by the structures **1c**.^5^ Class 1B and 1C rings are associated with only one 1,3-dipolar fragment in the ring. Structures **2** and **3** show examples of cross-conjugated (Class 2) and semiconjugated (Class 3) HMBs, respectively; these classes of HMB do not contain a 1,3-dipolar fragment.

As far as we are aware, the only monocyclic examples of Class 1A HMBs that have been characterized are 1,5-dithia-2,4,6,8-tetraazocine derivatives **4**, first reported by R.B. Woodward and co-workers in 1981.^6^ The diphenyl derivative **4** (*R* = Ph) (mp 225–6 °C) was shown by an X-ray analysis to be perfectly planar with bond lengths consistent with a delocalized aromatic 10π-electron ring. The derivatives **4** (*R* = 4-MeOC_6_H_4_ and 4-EtO_2_C.C_6_H_4_) have similar properties, but the dimethylamino derivative **4** (*R* = Me_2_N) was shown to be nonplanar. Subsequently, planar di-*tert*-butyl derivative **4** (*R* = tBu) was prepared by Gleiter and co-workers in 1984.^7^
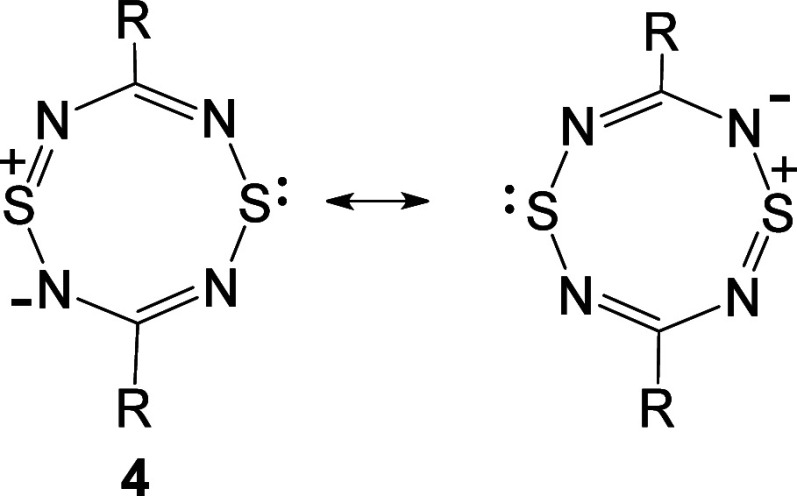


In the period 1984–1987, several theoretical studies of 1,5-dithia-2,4,6,8-tetraazocine derivatives **4**, employing *ab initio* and semiempirical methods, were reported.^8−10^ These studies primarily focused on the bonding relationship between the planar diphenyl derivative **4** (*R* = Ph) and the nonplanar dimethylamino derivative **4** (*R* = Me_2_N). A 2007 EPR spectro-electrochemistry study of the derivatives **4** (*R* = Ph, tBu) included DFT calculations.^11^ Broader investigations of this general family of Class 1A HMBs, of which the systems **1a** are the parent rings and which can be conveniently referred to as octabetaines, have not been reported. The known derivatives **4** appear to be thermally stable up to their melting points (**4**; *R* = Ph. mp 225–226 °C), but nothing is known about their photostability or chemical reactivity. We now describe the results of an investigation of the structures and properties of this neglected family of aromatic heterocycles.

## 2 Results and Discussion

### 2.1 Structure and Substituent Effects

We have calculated the energies, geometries, aromaticity indexes, and frontier orbitals of representative 1,5-dihydro-1,5-diazocines **5** (Table 1A), 1,5-dithiacines **6** (Table 1B), and 5-hydro-1,5-thiazocines **7** (Table 1C) at the B3LYP-D3/6-311++G(d,p) level of theory. In addition, we have investigated the influence of substituents on the structures of 2,4,6,8-substituted; 3,7-disubstituted; and 1,5-disubstituted dihydrodiazocines **8**, **9**, and **10** (Table 2).

**Table 1:**
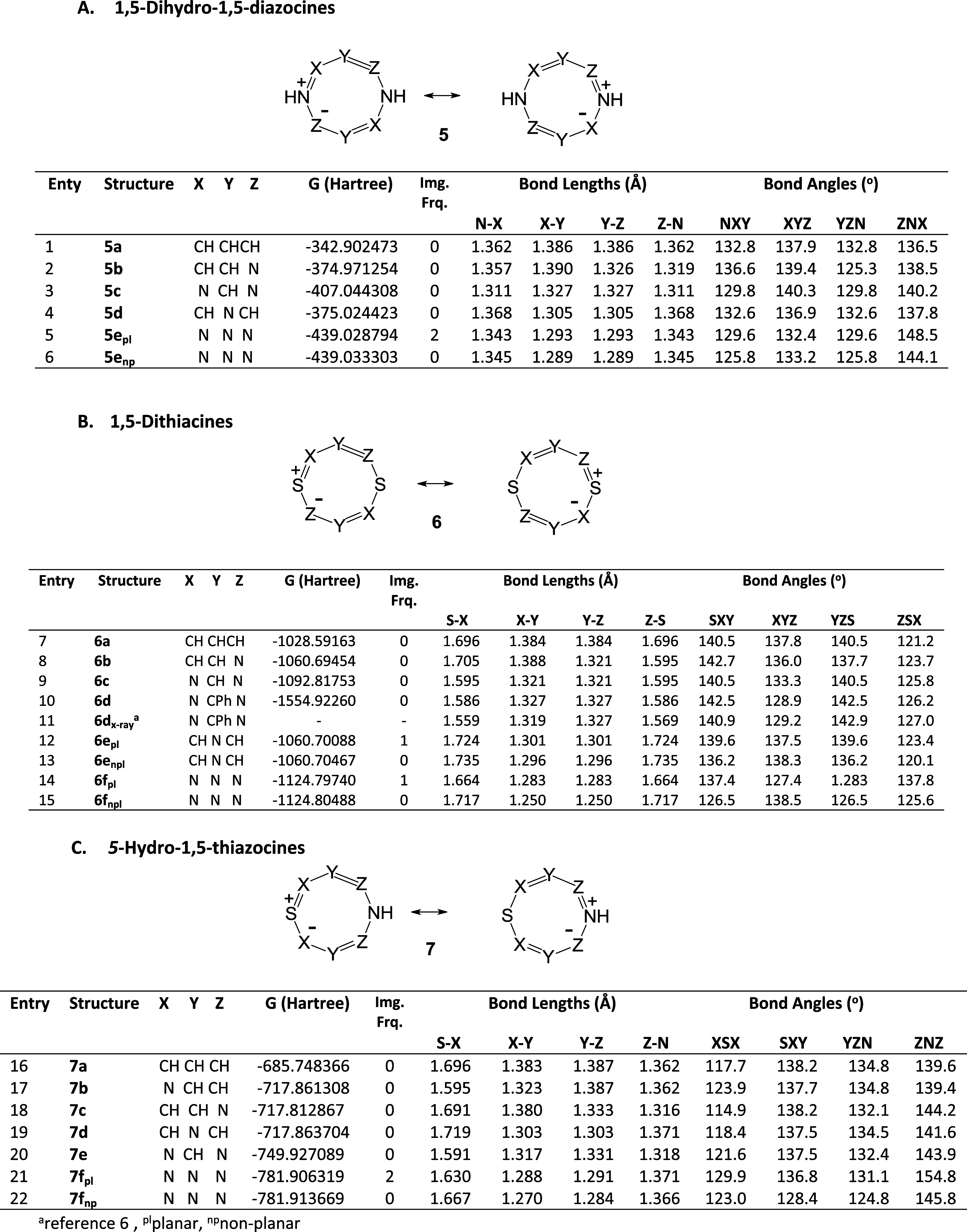
DFT Calculated Gibbs-Free Energies and Geometries of HMBs **5**, **6**, and **7**

a Ref (6); pl, planar; np, nonplanar

**Table 2:**
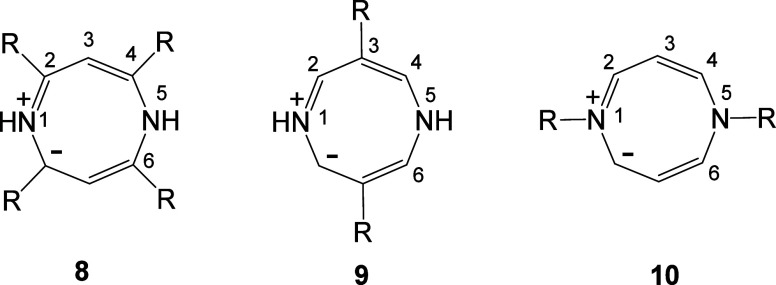

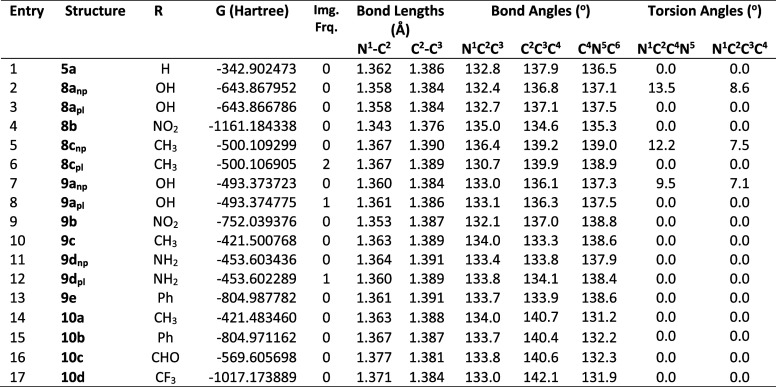
DFT Calculated Gibbs-Free Energies and Geometries of Substituted 1,5-Dihydro-1,5-diazocines **8**, **9**, and **10**^a^

a pl, planar; np, nonplanar

With four exceptions, the eight-membered HMBs summarized in Table 1 were calculated to be perfectly planar species with ring bond lengths consistent with cyclic conjugation. The calculated C–C bond lengths in the parent structures **5a**, **6a**, and **7a** (Table 1, entries 1, 7, and 16) are 1.385 ± 0.002 Å, which are comparable to the aromatic bond lengths in benzene (1.40 Å), pyrrole (1.38 Å), and thiophene (1.37 Å). The C–N bond lengths in structure **5a** (1.362 Å) and the C–S bond lengths in structure **6a** (1.692 Å) are comparable to those in pyrrole (1.375 Å) and thiophene (1.733 Å), respectively. The calculated structure of 3,7-diphenyl derivative **6d** (Table 1, entry 10) is in satisfactory agreement with the reported crystal structure (Table 1, entry 11). The properties of the four nonplanar species **5e**, **6e,f**, and **7f** are summarized in Table 3. These molecules adopt a twist conformation with an energy difference from the planar species in the range 2.4–4.7 kcal mol^–1^. This distortion from the planar configuration can be attributed to the minimization of repulsion between adjacent nitrogen lone pairs and minimization of angle strain.

**Table 3:**
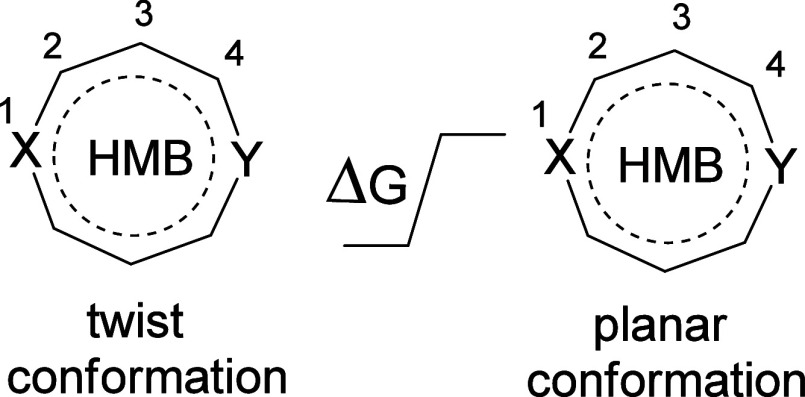

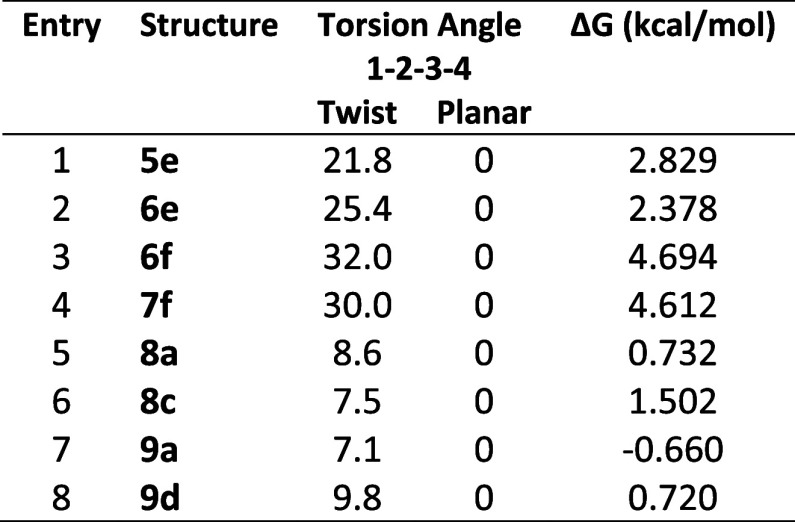
DFT Calculated Gibbs-Free Energy Differences (Δ*G*) of Planar and Nonplanar HMBs

Table 2 illustrates the effect of selected substituents on the 1,5-diazocine ring. In the tetrasubstituted derivatives **8**, π-donating substituents (OH, Me) (Table 2, entries 2, 3, 5, and 6) result in distortion to a nonplanar structure. Presumably, this is due to the increase of the π population of the ring. A similar effect by OH and NH_2_ substituents is observed in the disubstituted derivatives **9a** and **9d** (Table 2, entries 7, 8, 11, and 12). The substituents Me and Ph are weaker π donors, and in the disubstituted 1,5-diazocine derivatives **9c** and **9e** (Table 2, entries 10 and 13), the effect is not great enough to result in a distortion from planarity. This is also the case for the 1,5-dithiacine **6d** (Table 1, entries 10 and 11).

The structural properties of the four nonplanar substituted species **8a**,**c** and **9a,d** are summarized in Table 3 (entries 5–8). Compared to the structures **5e**, **6e,f**, and **7f** (Table 3, entries 1–4), the degree of twist in the nonplanar structures and the energy difference between planar and nonplanar conformations are smaller. Interestingly, the planar structure of derivative **8a** is an energy minimum but higher in energy than the twist conformation whereas planar **8c** is a second-order saddle point. For structure **9a** (Table 3, entry 7), the Gibbs-free energy is lower for the planar structure despite it actually being a transition state. This is also true for all other forms of energy, which include nuclear motions (E+ZPE, E-thermal, H). However, the total electronic energy of the planar structure **9a** is higher than that of the nonplanar, which indicates that on the potential energy hypersurface, the planar structure is indeed a transition state. This is because an extremum is qualified as a minimum or a transition state on the potential energy hypersurface (which corresponds to the total electronic energy). Only after that the corrections for nuclear motions are added. In most cases, the transition state is much higher in energy, and these corrections cannot make it lower, but this is a rare case where the energy of a transition state is very close to the neighboring minimum and the addition of corrections makes it lower in G.

The 1,5-disubstituted dihydrodiazocines **10** (*R* = CH_3_, Ph, CHO, CF_3_) (Table 2, entries 14–17) show an interesting structural variation when compared with the 1,5-NH derivatives **8**, **9**, and **5a**. For all four *N*,*N*-disubstituted derivatives, there is an elongation of the ring characterized by an increase in the C–C–C bond angles and a particularly significant reduction in the C–N–C bond angles (Table 2). This may be a steric effect; it is not an electronic effect as the selected substituents have opposite resonance and inductive characteristics.

### 2.2 Aromaticity and Bonding

Like the cyclooctatetraene dianion (COT^2–^), the HMBs **1a** have 10π electrons in cyclic conjugation, which corresponds to an aromatic ring according to Hückel’s [4*n* + 2] rule (*n* = 2). However, these π electrons are confined to an eight-membered ring (4*n*), which leads to a higher energy of the highest π bonding orbitals compared to those in, for example, benzene that has [4*n* + 2] π electrons in a [4*n* + 2] ring. The 10π cyclooctatetraene dianion has aromatic properties but is highly destabilized by Coulomb repulsion due to its negative charge. In the gas phase, COT^2–^ is highly unstable. It is of interest, therefore, to explore the aromatic character and bonding of neutral eight-membered isoelectronic HMBs **5**–**10**. In particular, we have evaluated the classical and magnetic aromaticity of selected HMBs using (i) the pEDA (pi electron donor–acceptor) index,^12^ (ii) the harmonic oscillator model of aromaticity (HOMA) index,^13,14^ (iii) aromatic stabilisation energy (ASE),^15^ (iv) the nucleus-independent chemical shifts (NICS(1)_*zz*_) index^16^ and (v) the anisotropy of induced current density (ACID) method.^17^

The pEDA index measures the π population of a ring and is the sum of the 2p_*z*_ atomic orbital population minus the aromatic [4*n* + 2] value, which in the case of HMBs **5**–**10** is 10. An inspection of Table 4 reveals that all the pEDA values for structures **5**–**7** are in the range −0.07 to −0.01, which is entirely consistent with a 10π system. Substituent effects (Table 5) modify the π population to a small extent with electron-withdrawing substituents such as NO_2_ (Table 5, entries 4 and 9) and CHO (Table 5, entry 16) increasing the pi-deficiency.

**Table 4:**
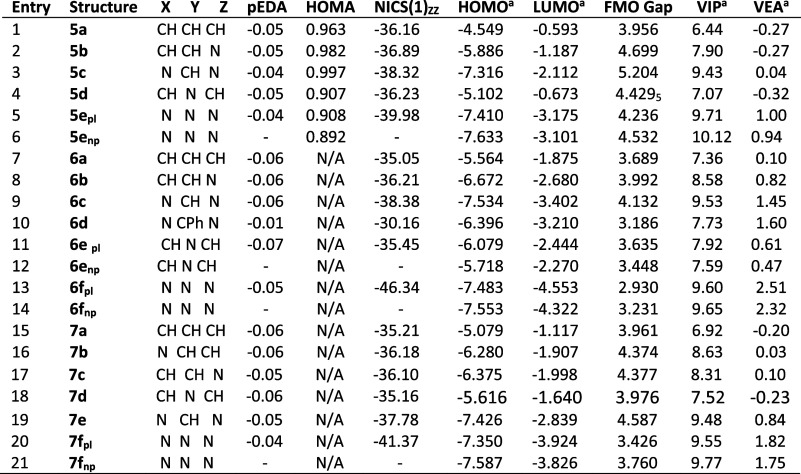
DFT Calculated Aromaticity Indices and Orbital Properties for HMBs **5**, **6**, and **7**

a Electron volts (eV); pl, planar; np, nonplanar

**Table 5:**
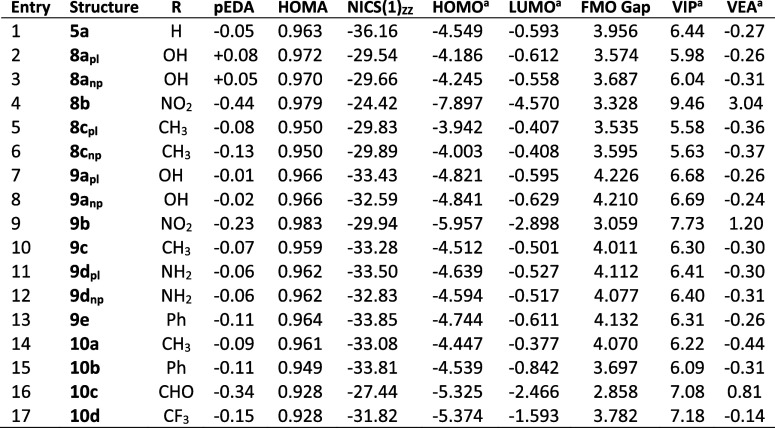
DFT Calculated Aromaticity Indices and Orbital Properties for HMBs **8**, **9**, and **10**

a Electron volts (eV); pl, planar; np, nonplanar

A measure of classical aromaticity is the geometry-based HOMA index. Calculated HOMA values for the planar derivatives **5a**–**e** are shown in Table 4 and are in the range of 0.91–0.99 (benzene HOMA = 1). This is indicative of a high degree of classical aromaticity and is consistent with the bond lengths reported in Section 2.1 (Tables 1A and 2). The HOMA values for substituted derivatives **8**–**10** vary in the range 0.95–0.98. The HOMA model has not been parametrized for sulfur heterocycles, and values for structures **6** and **7** are not available.

**Scheme 1:**
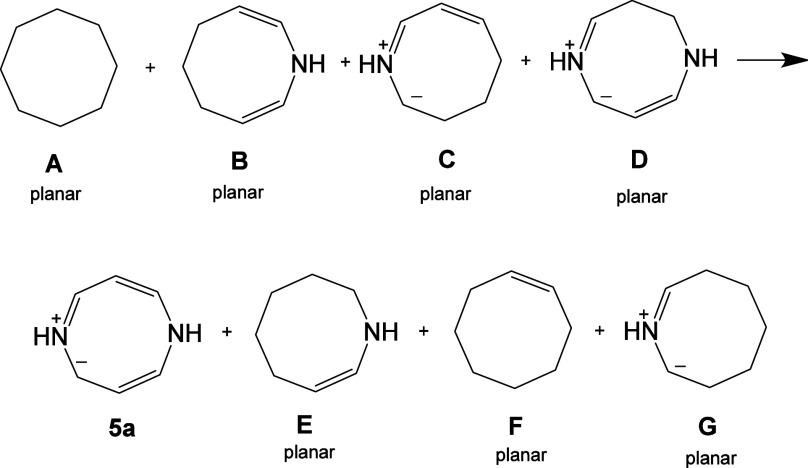
Homodesmotic Scheme Used to Calculate the Aromatic Stabilization Energy of Dihydrodiazocine **5a**

An energy-based measure of aromaticity is ASE, which measures the extra stabilization due to cyclic conjugation. Employing the approach described by Schleyer and co-workers,^15^ we have used the homodesmotic reaction shown in Scheme 1 to estimate the ASE of HMB **5a**. The structures **A**–**G** in Scheme 1 are constrained to planarity to eliminate variation in the conformational energy. The gas-phase optimized energies including zero-point vibrational correction (ZPE) of structures **A**–**G** and **5a** are shown in Table 6. Based on these calculated energies, Scheme 1 gives an ASE value of −26.8 kcal mol^–1^ for ring **5a**. This is comparable to the values of −25 to −26 kcal mol^–1^ calculated for COT^2–^ by Sokolov and co-workers^18^ and is similar to the value of −33 kcal mol^–1^ calculated for benzene. All these values are best regarded as estimates, but the ASE value of −26.8 kcal mol^–1^ for structure **5a** does suggest that these betaines enjoy significant stabilization associated with cyclic conjugation, and this, combined with the observed HOMA values, suggests that these betaines are associated with a classical aromatic character.

**Table 6:**
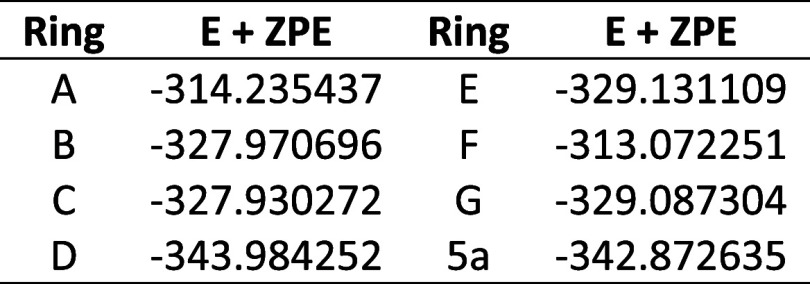
Gas-Phase Optimized Energies Including the ZPE of Planar Structures **A**–**G** and **5a** Calculated at the B3LYP-D3/6-311++G(d,p) Level of Theory

The concept of aromaticity has broaden to many structural types, and even the usefulness of the term has been questioned.^19^ However, the concept originally evolved to describe small planar rings with cyclic conjugation, and for these molecules, two discrete definitions of aromaticity are generally accepted: classical aromaticity, characterized by structure and energy, is considered to be a separate property to magnetic aromaticity.^15^ A measure of magnetic aromaticity is the NICS(1)_*zz*_ index. More negative values indicate higher magnetic aromaticity; the value for benzene using the B3LYP-D3/6-311++G(d,p) model is −29.3. Calculated NICS(1)_*zz*_ values for HMBs **5**, **6**, and **7** are shown in Table 4. The parent rings **5a**, **6a**, and **7a** have NICS(1)_*zz*_ values of −36.2, −35.1, and −35.2 (Table 4, entries 1, 7, and 15), which are indicative of a strong ring current and high degree of magnetic aromaticity. Relative to the parent rings, tetraza substitution in the 2, 4, 6, and 8 positions lowers NICS(1)_*zz*_ by 2–3 units (Table 4, entries 3, 9, and 19) whereas 3,7-diazasubstitution has little effect (Table 4, entries 4, 11, and 18). In the planar structures of the fully aza derivatives (Table 4, entries 5, 13, and 20), a much larger effect in the range −4 to −11 is observed. The planar structure **6f** (Table 4, entry 13) has a NICS(1)_*zz*_ value of −46.3, which is exceptionally large. The reason for this is not clear, but it may be associated with conjugation of the lone pairs around the perimeter of the ring; this is maximized in the 1,5-dithiacine **6f**.

**Figure 1:**
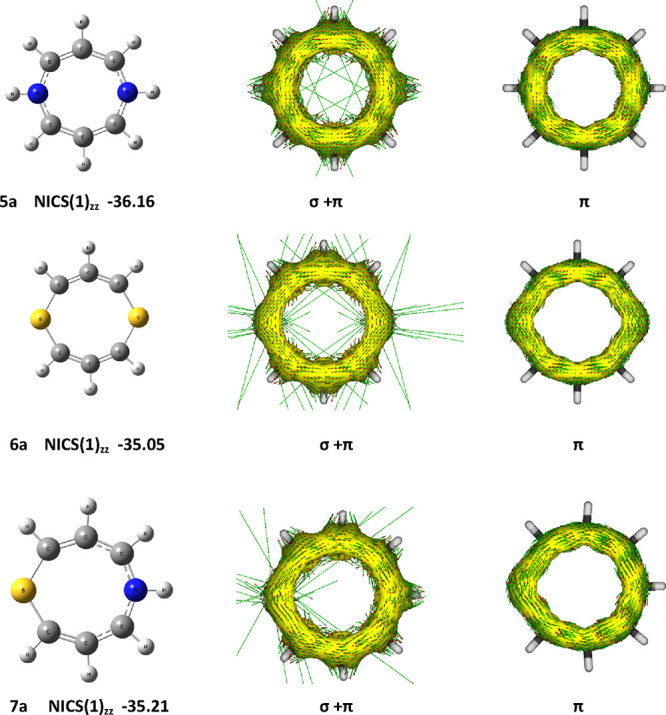
ACID σ + π and π electron current density maps for rings **5a**, **6a**, and **7a**.

NICS values have been widely used to characterize the magnetic aromaticity of heterocycles. However, their use without an accompanying analysis of current density maps to demonstrate the presence of a ring current has been questioned.^20,21^ To address this, we have applied the ACID method to investigate electron delocalization in the parent rings **5a**, **6a**, and **7a**. The observed σ + π and π electron current density maps for rings **5a**, **6a**, and **7a** are shown in Figure 1. The π electron density maps are consistent with high π cyclic conjugation suggested by the high NICS(1)_*zz*_ values, and we conclude that the NICS(1)_*zz*_ values do reflect the magnitudes of the π-electron ring currents and magnetic aromaticity of this class of heterocycle.

### 2.3 Frontier Orbitals and Reactivity

Calculated frontier orbital energies (HOMO and LUMO) and calculated VIPs and VEAs for the betaines **5a**–**e**, **6a**–**e**, and **7e**–**f** are shown in Table 4. These derivatives enjoy a significant classical and magnetic aromatic character (Section 2.2). The 1,5-diazocine **5a** has an estimated ASE of −26.8 kcal mol^–1^ ; however, this derivative also has a high-energy HOMO (−4.549 eV) and a low VIP (6.4 eV) (Table 4, entry 1), which are properties that might be associated with an antiaromatic character and high reactivity. Due to this unusual electronic profile, the bonding and reactivity of octabetaines **5**–**7** merit closer examination.

**Figure 2:**
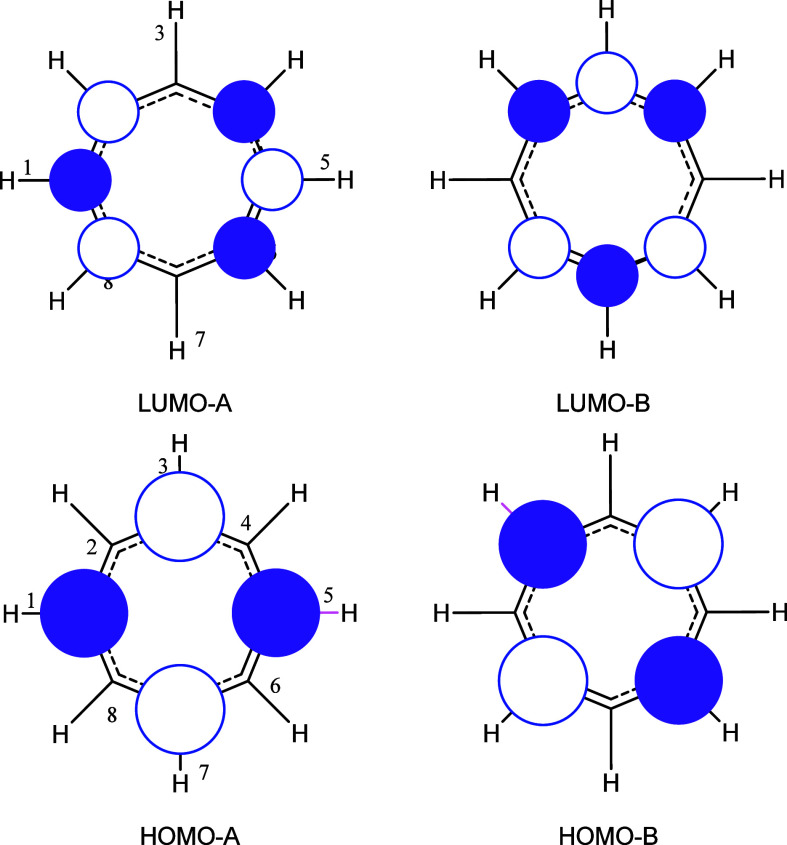
Degenerate pairs of frontier orbitals of C_8_H_8_^2–^.

The calculated VIP values in Table 4 vary in the range 6.4–10.1 eV. This large variation can be understood by considering the heterocycles **5**–**7** as intramolecular perturbations^22^ of the isoelectronic cyclooctatetraene dianion (C_8_H_8_^2–^). This dianion is characterized by a pair of doubly occupied degenerate highest occupied frontier orbitals that equate to two nonbonding molecular orbitals (HOMO-A and HOMO-B), each with two nodes perpendicular to the ring and having the forms shown in Figure 2. Based on these orbital maps, heteroatom perturbation at positions 1, 3, 5, and 7 of the ring can be expected to lower the energy of HOMO-A and have a minimal effect on HOMO-B. Alternatively, heteroatom perturbation at positions 2, 4, 6, and 8 will lower the energy of HOMO-B but have a minimal effect on HOMO-A. This perturbation model satisfactorily rationalizes the variation in calculated orbital properties shown in Table 4 and is illustrated as follows by application to the dihydrodiazocines **5**.

**Figure 3:**
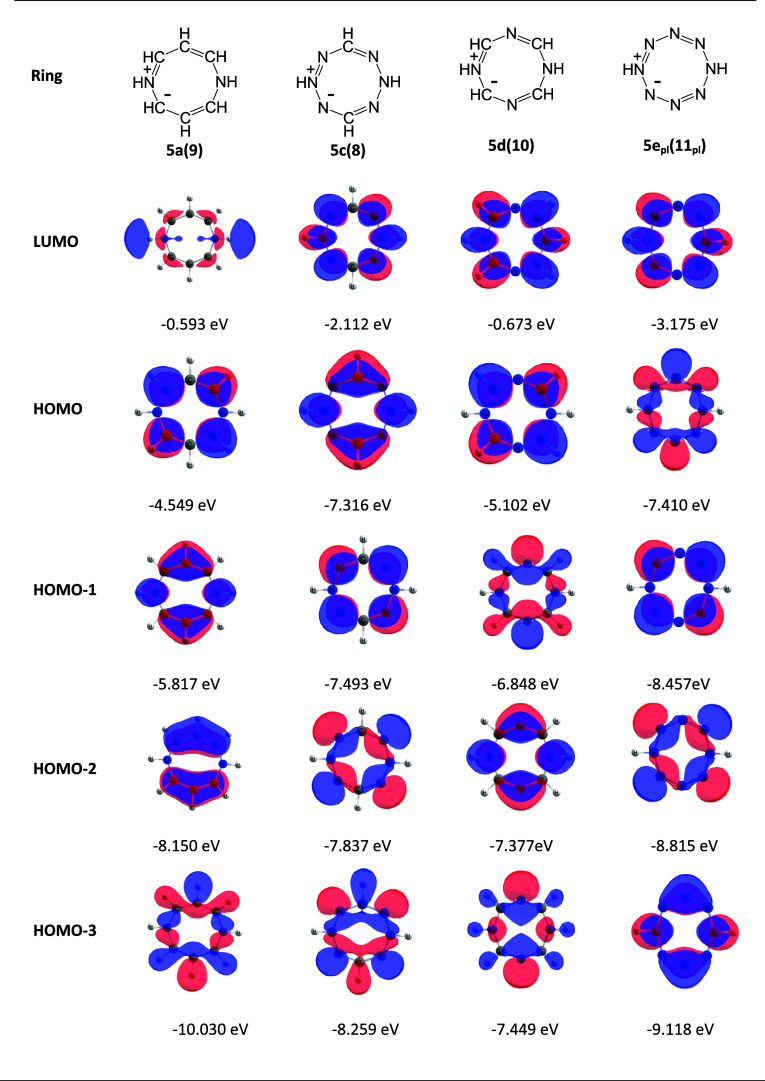
B3LYP-D3/6-311++G(d,p) calculated orbitals of rings **5a**, **5c**, **5d**, and **5e**.

The calculated frontier orbital maps of the dihydrodiazocines **5a**, **5c**, **5d**, and **5e** are shown in Figure 3. Consider the orbitals calculated for parent ring **5a** (Figure 3, column 1). The HOMO is high in energy and topologically corresponds to HOMO-B (Figure 2) with nodes at positions 1, 3, 5, and 7; the NH groups at positions 1 and 5 are at nodal positions and do not perturb HOMO-B. In contrast, the NH groups do perturb HOMO-A, leading to the spatially related and lower energy HOMO-1 for ring **5a**. Unexpectedly, the LUMO of ring **5a** is an σ-orbital, and the first unoccupied π-orbital has an energy of −0.243 eV.

In ring **5c** (Figure 3, column 2), the perturbation of HOMO-B by four nitrogen at positions 2, 4, 6, and 8 results in significant lowering of the energy of the corresponding molecular orbital to the position of HOMO-1. In this ring, the 1,5-NH groups also lower HOMO-A, but not as much as HOMO-B, and this orbital now becomes the HOMO of **5c**, resulting in a much higher VIP (9.43 eV) for **5c** compared to that of **5a** (6.44 eV). The HOMO and HOMO-1 of ring **5c** are both lower in energy than those of ring **5a**, and the orbital topologies have been interchanged (Figure 3, columns 2 and 3).

In ring **5d** (Figure 3, column 3), the four nitrogen (2N + 2NH) at positions 1, 3, 5, and 7 substantially perturb HOMO-A leading it to becoming HOMO-2 for this molecule. However, HOMO-B is unaffected, and the HOMO of ring **5d** is closer in energy and topology to that of ring **5a**. In ring **5e** (planar) (Figure 3, column 4), with eight nitrogen (6N + 3NH), there is perturbation of both HOMO-A and HOMO-B leading to their transformation from HOMOs to the positions of HOMO-1 and HOMO-3.

The variation in the energies of the LUMOs and VEAs is similarly rationalized as perturbations of the degenerate pair of C_8_H_8_^2–^ LUMOs, each with three nodes perpendicular to the plane of the ring (LUMO-A and LUMO-B, Figure 2). For the dihydrodiazocines **5**, the 1,5-nitrogen atoms lower the energy of LUMO-A with little effect on LUMO-B; the first π-LUMO orbital energy of ring **5a** is −0.245 eV. The replacement of CH by N at positions 2, 4, 6, and 8 results in significant lowering of LUMO-A, as seen in the LUMO energies and topology of rings **5c** and **5e** (Figure 3). Aza groups at positions 3 and 7 can be expected to have little effect on LUMO-A as they are at the nodal positions. However, the inductive effect on the neighboring carbon atoms combined with the effect of the 1,5-NH groups results in a LUMO energy for ring **5d** below that of ring **5a**, but still much higher in energy than those of rings **5c** and **5e**.

A similar approach accounts for the variation in the frontier orbital energies, VIPs, and VEAs of the 1,5-dithiacines **6** and 5-hydro-1,5-thiazocines **7** (Table 4). Using this perturbation model, the orbital energy levels, and in particular HOMO energies and VIPs, of rings **5**–**7** can be adjusted to approach a desired value by selective positioning and number of ring nitrogen atoms.

## 3 Conclusions

The octabetaines **5**–**7** constitute a large family of heterocycles that have received little attention. Calculated properties (HOMA, ASE) suggest that they enjoy stabilization associated with cyclic conjugation and have some classical aromatic character (10π). A few examples of 2,4,6,8-tetraaza-1,5-dithicines **6** are known and are planar, stable heterocycles. Based on calculated properties, other examples of rings **5**–**7** can also be expected to be stable. Electron-donating substituents result in distortion to a nonplanar geometry due to an increase in the π content of the ring.

The rings **5**–**7** are [4*n* + 2] π-electron heterocycles (10π) with [4*n*] component atom rings (8). This has the consequence that the highest occupied orbitals are much higher in energy than those in classical aromatic rings (e.g., benzene, pyrrole), and this is an uncharacteristic property of aromatic rings. High-energy HOMOs are usually associated with antiaromatic rings. This unusual combination of electronic properties is of particular interest and may potentially lead to compounds with useful applications. The HOMO and LUMO energy levels can be predictably adjusted by the judicious choice of the ring atoms and substituents.

The rings **5**–**7** also show high levels of magnetic aromaticity as measured by the values of their NICS(1)_*zz*_ index and current density maps. The heterocycles **5**–**7** have NICS(1)_*zz*_ values typically in the range −36 to −38 with exceptional values > −40. These high values of magnetic properties may also have useful applications.

Heterocyclic chemistry is a mature and widely explored area of chemistry; its origins go back to the 1830s and the isolation of pyrrole from coal tar. In assessing an area of study, it is important to know what is not known as well as what is known. There are areas of heterocyclic chemistry that remain largely unexplored. We have previously drawn attention to an interesting class of six-membered heterocycles for which a single derivative (stable and crystalline) has been reported.^23,24^ In this study, we draw attention to a larger class of eight-membered heterocycles, and on the basis of DFT calculations, useful properties for judiciously selected derivatives can be predicted.

## 4 Computational Details

All calculations were performed using the Gaussian 16 suite of programs.^25^ Hybrid functional B3LYP^26,27^ with Grimme dispersion correction (B3LYP-D3)^28^ was used in conjunction with triple-ζ Pople basis set 6-311++G(d,p).^29,30^ All geometry optimizations were followed by frequency calculations to establish the nature of the stationary points and to calculate the ZPE and thermal corrections to the Gibbs-free energy. The number of imaginary frequencies of structures being saddle points is reported in the text. VIP and VEA were calculated as the energy difference between a neutral molecule and a positive/negative ion with the same molecular geometry.^31^ Three aromaticity indices were calculated: geometric HOMA,^13,14^ magnetic NICS(1)_ZZ_,^32,33^ and electronic pEDA.^12^ The NICS(1)_ZZ_ index was calculated as the z-component(perpendicular) of shielding constant of a ghost atom laying 1 Å above the geometric center of the ring. ACID maps were calculated by using the software package developed by Herges and Geuenich.^34^

## Data Availability

The data underlying this study are available in the published article and its Supporting Information.

## Supporting Information Available

Calculated structures (Cartesian coordinates) for all molecules; Gibbs-free energies (Hartrees); number of imaginary frequencies (PDF)
