# Supplementary material for: Octabetaines: a DFT Study of Unexplored Eight-Membered 10π Heterocycles
Source: ACS Omega. 2025 Jan 28;10(5):4978–86. doi: 10.1021/acsomega.4c10544 (PMC11822512; doi:10.1021/acsomega.4c10544)
Supplement: Supplementary file 1 — ao4c10544_si_001.pdf [file ao4c10544_si_001.pdf]

Supporting Information

Octabetaines: A DFT Study of Unexplored Eight-Membered 10π Heterocycles

Christopher A. Ramsden[a]* and Wojciech P. Oziminski[b]*

[a]Lennard-Jones Laboratories, School of Chemical and Physical Sciences, Keele University, Staffordshire ST5 5BG, UK

[b]Department of Organic and Physical Chemistry, Faculty of Pharmacy, Medical University of Warsaw, 1 Banacha Street,02-097 Warsaw, Poland

Table of Contents for Supporting Information only

Table S1A. Structures of 1,5-Dihydro-1,5-diazocines type **5** S2

Table S1B. Structures of 1,5-Dithiacines type **6**....S6

Table S1C. Structures of 5-Hydro-1,5-thiazocines type **7** S12

Table S2. Structures of 1,5-dihydro-1,5-diazocines type 8 - 10 ....S17

Table S1A. Structures of 1,5-Dihydro-1,5-diazocines type **5**

5a

	angstroms		
	X	Y	Z
N	-0.000391000	1.798806000	0.000000000
C	-1.264588000	1.293094000	0.000000000
C	1.761783000	0.000556000	0.000000000
C	-1.761783000	-0.000556000	0.000000000
C	1.264588000	-1.293094000	0.000000000
N	0.000391000	-1.798806000	0.000000000
H	-0.001615000	2.807451000	0.000000000
H	0.001615000	-2.807451000	0.000000000
H	-2.004263000	2.083190000	0.000000000
H	-2.847504000	-0.000027000	0.000000000
H	2.004263000	-2.083190000	0.000000000
H	2.847504000	0.000027000	0.000000000
C	1.264588000	1.294247000	0.000000000
C	-1.264588000	-1.294247000	0.000000000
H	-2.003144000	-2.085440000	0.000000000
H	2.003144000	2.085440000	0.000000000

Gibbs free energy = -342.902473 Hartree

Number of imaginary frequencies: 0

5b

	angstroms		
	X	Y	Z
N	1.276238000	1.195732000	0.000000000
C	0.009147000	1.682354000	0.000000000
N	1.884138000	0.025124000	0.000000000
C	1.276238000	-1.153681000	0.000000000
C	-1.276238000	1.153681000	0.000000000
C	-0.009147000	-1.682354000	0.000000000
N	-1.884138000	-0.025124000	0.000000000
N	-1.276238000	-1.195732000	0.000000000
H	1.973752000	1.926972000	0.000000000
H	-1.973752000	-1.926972000	0.000000000
H	0.032677000	2.765471000	0.000000000
H	-2.021794000	1.943694000	0.000000000
H	-0.032677000	-2.765471000	0.000000000
H	2.021794000	-1.943694000	0.000000000

Gibbs free energy = -374.971254 Hartree

Number of imaginary frequencies: 0

5c

	angstroms		
	X	Y	Z
N	0.000000000	-1.694267000	0.000000000
N	-1.232753000	-1.247942000	0.000000000
N	1.232753000	-1.247942000	0.000000000

C	1.683260000	0.000000000	0.000000000
C	-1.683260000	0.000000000	0.000000000
N	1.232753000	1.247942000	0.000000000
N	-1.232753000	1.247942000	0.000000000
N	0.000000000	1.694267000	0.000000000
H	0.000000000	-2.706143000	0.000000000
H	0.000000000	2.706143000	0.000000000
H	-2.768273000	0.000000000	0.000000000
H	2.768273000	0.000000000	0.000000000

Gibbs free energy = -407.044308 Hartree

Number of imaginary frequencies: 0

5d

	angstroms		
	X	Y	Z
N	0.000000000	-1.706137000	0.000000000
C	1.276340000	-1.213956000	0.000000000
C	-1.276340000	1.213956000	0.000000000
N	0.000000000	1.706137000	0.000000000
H	0.000003000	-2.718198000	0.000000000
H	-0.000003000	2.718198000	0.000000000
H	2.006826000	-2.016836000	0.000000000
H	-2.006826000	2.016836000	0.000000000
C	-1.276340000	-1.213959000	0.000000000
C	1.276340000	1.213959000	0.000000000
H	2.006823000	2.016842000	0.000000000

H	-2.006823000	-2.016842000	0.000000000
N	1.755695000	0.000000000	0.000000000
N	-1.755695000	0.000000000	0.000000000

Gibbs free energy = -375.024423 Hartree

Number of imaginary frequencies: 0

5e_pl

	angstroms		
	X	Y	Z
N	0.000000000	0.000000000	1.547185000
N	0.000000000	1.292545000	1.182864000
N	0.000000000	-1.292545000	1.182864000
N	0.000000000	-1.292545000	-1.182864000
N	0.000000000	1.292545000	-1.182864000
N	0.000000000	0.000000000	-1.547185000
H	0.000000000	0.000000000	2.561042000
H	0.000000000	0.000000000	-2.561042000
N	0.000000000	1.814614000	0.000000000
N	0.000000000	-1.814614000	0.000000000

Gibbs free energy = -439.028794 Hartree

Number of imaginary frequencies: 2

5e_np

	angstroms		
	X	Y	Z
N	0.000000000	0.000000000	-1.556188000
N	1.241670000	0.308271000	-1.142238000
N	-1.241670000	-0.308271000	-1.142238000
N	-1.241670000	0.308271000	1.142238000
N	1.241670000	-0.308271000	1.142238000
N	0.000000000	0.000000000	1.556188000
H	0.000000000	0.000000000	-2.569056000
H	0.000000000	0.000000000	2.569056000
N	1.753786000	0.000000000	0.000000000
N	-1.753786000	0.000000000	0.000000000

Gibbs free energy = -439.033303 Hartree

Number of imaginary frequencies: 0

Table S1B. Structures of 1,5-Dithiacines type **6**

6a

	angstroms		
	X	Y	Z
C	0.000000000	0.000000000	1.975455000
C	0.000000000	0.000000000	-1.975455000
H	0.000000000	0.000000000	-3.063282000
H	0.000000000	0.000000000	3.063282000
S	0.000000000	2.124249000	0.000000000
S	0.000000000	-2.124249000	0.000000000

C	0.000000000	-1.291274000	1.477455000
H	0.000000000	-2.073694000	2.230207000
C	0.000000000	1.291274000	-1.477455000
H	0.000000000	2.073694000	-2.230207000
C	0.000000000	1.291274000	1.477455000
H	0.000000000	2.073694000	2.230207000
C	0.000000000	-1.291274000	-1.477455000
H	0.000000000	-2.073694000	-2.230207000

Gibbs free energy = -1028.591627 Hartree

Number of imaginary frequencies: 0

6b

	angstroms		
	X	Y	Z
N	0.853683000	1.796215000	0.000000000
C	-1.771873000	-0.846216000	0.000000000
C	1.771873000	0.846216000	0.000000000
N	-0.853683000	-1.796215000	0.000000000
H	2.774433000	1.272459000	0.000000000
H	-2.774433000	-1.272459000	0.000000000
S	0.738130000	-1.897253000	0.000000000
S	-0.738130000	1.897253000	0.000000000
C	-1.771873000	0.541802000	0.000000000
H	-2.768277000	0.982064000	0.000000000
C	1.771873000	-0.541802000	0.000000000
H	2.768277000	-0.982064000	0.000000000

Gibbs free energy = -1060.694535 Hartree

Number of imaginary frequencies: 0

6c

	angstroms		
	X	Y	Z
N	0.000000000	1.213174000	1.419762000
N	0.000000000	1.213174000	-1.419762000
C	0.000000000	0.000000000	-1.943148000
C	0.000000000	0.000000000	1.943148000
N	0.000000000	-1.213174000	-1.419762000
N	0.000000000	-1.213174000	1.419762000
H	0.000000000	0.000000000	3.031486000
H	0.000000000	0.000000000	-3.031486000
S	0.000000000	-1.940499000	0.000000000
S	0.000000000	1.940499000	0.000000000

Gibbs free energy = -1092.817526 Hartree

Number of imaginary frequencies: 0

6d

	angstroms		
	X	Y	Z
C	1.987329000	0.000004000	-0.000012000
C	-1.987269000	0.000004000	-0.000027000
S	0.000050000	-1.914566000	-0.000535000

N	1.414465000	-1.197146000	-0.010938000
N	-1.414537000	-1.197213000	0.010180000
N	-1.414536000	1.197206000	-0.010217000
N	1.414462000	1.197139000	0.010918000
S	0.000049000	1.914572000	0.000512000
C	3.485908000	-0.000001000	0.000000000
C	4.197725000	1.203806000	-0.084038000
C	4.197721000	-1.203806000	0.084050000
C	5.589368000	1.201755000	-0.085363000
H	3.652866000	2.136397000	-0.148381000
C	5.589367000	-1.201756000	0.085397000
H	3.652865000	-2.136399000	0.148385000
C	6.291344000	-0.000002000	0.000022000
H	6.126212000	2.141274000	-0.153290000
H	6.126208000	-2.141277000	0.153331000
H	7.375546000	-0.000001000	0.000030000
C	-3.485917000	-0.000001000	-0.000007000
C	-4.197743000	1.203860000	0.083071000
C	-4.197745000	-1.203861000	-0.083065000
C	-5.589390000	1.201821000	0.084363000
H	-3.652894000	2.136506000	0.146694000
C	-5.589394000	-1.201821000	-0.084322000
H	-3.652900000	-2.136509000	-0.146702000
C	-6.291378000	-0.000001000	0.000029000
H	-6.126221000	2.141413000	0.151413000
H	-6.126224000	-2.141414000	-0.151358000
H	-7.375579000	0.000001000	0.000043000

Gibbs free energy = -1554.922604 Hartree

Number of imaginary frequencies: 0

6e_pl

angstroms

	X	Y	Z
S	-2.030576000	-0.000001000	-0.000179000
S	2.030496000	0.000001000	-0.000143000
N	0.000017000	-1.989422000	0.000164000
N	0.000015000	1.989423000	0.000160000
C	1.212831000	-1.517382000	0.000098000
H	2.010230000	-2.258697000	-0.000015000
C	-1.212748000	-1.517511000	0.000111000
H	-2.010184000	-2.258774000	0.000187000
C	1.212830000	1.517382000	0.000092000
H	2.010227000	2.258699000	-0.000027000
C	-1.212751000	1.517509000	0.000120000
H	-2.010185000	2.258774000	0.000211000

Gibbs free energy = -1060.700877 Hartree

Number of imaginary frequencies: 1

6e_np

angstroms

	X	Y	Z
S	-2.036586000	0.000000000	0.000019000
S	2.036589000	-0.000006000	0.000017000

N	0.000000000	-1.932139000	-0.000025000
N	-0.000003000	1.932133000	-0.000003000
C	1.170349000	-1.470169000	-0.312391000
H	1.868757000	-2.164218000	-0.772687000
C	-1.170342000	-1.470174000	0.312375000
H	-1.868769000	-2.164257000	0.772591000
C	1.170347000	1.470180000	0.312364000
H	1.868831000	2.164324000	0.772398000
C	-1.170357000	1.470163000	-0.312377000
H	-1.868810000	2.164279000	-0.772502000

Gibbs free energy = -1060.704668 Hartree

Number of imaginary frequencies: 0

6f_pl

	angstroms		
	X	Y	Z
N	0.000000000	1.552439000	1.150078000
N	0.000000000	-1.552439000	1.150078000
N	0.000000000	-1.552439000	-1.150078000
N	0.000000000	1.552439000	-1.150078000
S	0.000000000	0.000000000	-1.748764000
S	0.000000000	0.000000000	1.748764000
N	0.000000000	2.120474000	0.000000000
N	0.000000000	-2.120474000	0.000000000

Gibbs free energy = -1124.797401 Hartree

Number of imaginary frequencies: 1

6f_np

	angstroms		
	X	Y	Z
N	-1.074674000	1.456127000	0.460614000
N	-1.074674000	-1.456127000	-0.460614000
N	1.074674000	-1.456127000	0.460614000
N	1.074674000	1.456127000	-0.460614000
S	1.859796000	0.000000000	0.000000000
S	-1.859796000	0.000000000	0.000000000
N	0.000000000	1.898882000	0.000000000
N	0.000000000	-1.898882000	0.000000000

Gibbs free energy = -1124.804880 Hartree

Number of imaginary frequencies: 0

Table S1C. Structures of 5-Hydro-1,5-thiazocines type **7**

7a

	angstroms		
	X	Y	Z
C	0.000000000	1.765998000	0.000000000
C	0.797864000	-1.705311000	0.000000000
C	-1.317139000	1.345256000	0.000000000
C	-0.567868000	-1.948732000	0.000000000
N	-1.682673000	-1.166590000	0.000000000
H	-2.512252000	-1.741764000	0.000000000

H	0.152340000	2.840331000	0.000000000
H	-2.003672000	2.188039000	0.000000000
H	-0.842513000	-2.995737000	0.000000000
H	1.346321000	-2.643826000	0.000000000
C	1.653880000	-0.619422000	0.000000000
C	-2.024113000	0.151683000	0.000000000
H	-3.100934000	0.261723000	0.000000000
H	2.713404000	-0.853515000	0.000000000
S	1.548142000	1.073378000	0.000000000

Gibbs free energy = -685.748366 Hartree

Number of imaginary frequencies: 0

7b

	angstroms		
	X	Y	Z
C	0.900205000	-1.635721000	0.000000000
C	-1.270323000	1.368325000	0.000000000
C	-0.460077000	-1.908743000	0.000000000
N	-1.591166000	-1.149562000	0.000000000
H	-2.409853000	-1.741142000	0.000000000
H	-1.949476000	2.219771000	0.000000000
H	-0.701601000	-2.963938000	0.000000000
H	1.495211000	-2.547958000	0.000000000
C	-1.956786000	0.162661000	0.000000000
H	-3.034448000	0.264714000	0.000000000
S	1.432282000	1.034844000	0.000000000

N 1.649099000 -0.545456000 0.000000000

N 0.000000000 1.736720000 0.000000000

Gibbs free energy = -717.861308 Hartree

Number of imaginary frequencies: 0

7c

	angstroms		
	X	Y	Z
C	0.000000000	1.716680000	0.000000000
C	0.692293000	-1.701188000	0.000000000
C	-1.310958000	1.286316000	0.000000000
N	-1.655540000	-1.110090000	0.000000000
H	-2.496033000	-1.673694000	0.000000000
H	0.120088000	2.794943000	0.000000000
H	-2.008339000	2.121103000	0.000000000
H	1.199803000	-2.663293000	0.000000000
C	1.588184000	-0.651801000	0.000000000
H	2.631315000	-0.950038000	0.000000000
S	1.549527000	1.039054000	0.000000000
N	-0.621710000	-1.924582000	0.000000000
N	-2.016518000	0.155541000	0.000000000

Gibbs free energy = -717.812867 Hartree

Number of imaginary frequencies: 0

7d

	angstroms		
	X	Y	Z
C	0.000000000	1.747121000	0.000000000
C	0.570842000	-1.892297000	0.000000000
N	1.643825000	-1.038558000	0.000000000
H	2.499817000	-1.579381000	0.000000000
H	-0.201575000	2.816531000	0.000000000
H	0.912281000	-2.923005000	0.000000000
C	-1.577956000	-0.750243000	0.000000000
C	1.954136000	0.297057000	0.000000000
H	3.031593000	0.431325000	0.000000000
H	-2.630323000	-1.027386000	0.000000000
S	-1.532863000	0.968475000	0.000000000
N	-0.722850000	-1.733317000	0.000000000
N	1.255007000	1.397088000	0.000000000

Gibbs free energy = -717.863704 Hartree

Number of imaginary frequencies: 0

7e

	angstroms		
	X	Y	Z
C	0.794718000	-1.641625000	0.000000000
C	-1.262508000	1.316300000	0.000000000
N	-1.564713000	-1.088154000	0.000000000
H	-2.395981000	-1.666331000	0.000000000
H	-1.954913000	2.154190000	0.000000000

H	1.339189000	-2.582391000	0.000000000
S	1.430470000	0.994859000	0.000000000
N	-0.514322000	-1.883856000	0.000000000
N	-1.945206000	0.173440000	0.000000000
N	1.585801000	-0.588624000	0.000000000
N	0.000000000	1.691300000	0.000000000

Gibbs free energy = -749.927089 Hartree

Number of imaginary frequencies: 0

7f_pl

	angstroms		
	X	Y	Z
N	-1.715693000	0.000000000	0.000000000
H	-2.729010000	0.000000000	0.000000000
S	1.605639000	0.000000000	0.000000000
N	-0.281390000	1.953158000	0.000000000
N	-0.281390000	-1.953157000	0.000000000
N	-1.416444000	-1.337568000	0.000000000
N	-1.416444000	1.337567000	0.000000000
N	0.915593000	-1.476354000	0.000000000
N	0.915593000	1.476353000	0.000000000

Gibbs free energy = -781.906319 Hartree

Number of imaginary frequencies: 2

7f_np

	angstroms		
	X	Y	Z
N	-1.761930000	-0.000002000	0.000001000
H	-2.774193000	-0.000001000	-0.000007000
S	1.641060000	0.000003000	0.000000000
N	-0.282372000	1.823037000	0.024430000
N	-0.282366000	-1.823038000	-0.024431000
N	-1.359887000	-1.239859000	-0.407898000
N	-1.359890000	1.239855000	0.407899000
N	0.845885000	-1.412315000	0.389624000
N	0.845880000	1.412317000	-0.389625000

Gibbs free energy = -781.913669 Hartree

Number of imaginary frequencies: 0

Table S2. Structures of 1,5-dihydro-1,5-diazocines type 8 - 10

8a_np

	angstroms		
	X	Y	Z
N	-1.778674000	0.000122000	0.000098000
C	-1.281679000	1.259521000	0.107881000
C	-0.000130000	-1.769237000	-0.000117000
C	0.000144000	1.769231000	-0.000047000
C	1.281688000	-1.259499000	0.107917000
N	1.778679000	-0.000136000	-0.000027000
H	-2.792235000	0.000203000	0.000187000
H	2.792239000	-0.000184000	0.000231000

H	0.000196000	2.853440000	-0.000150000
H	-0.000171000	-2.853441000	-0.000261000
C	-1.281880000	-1.259315000	-0.107891000
C	1.281905000	1.259349000	-0.107895000
O	-2.338089000	-2.169549000	-0.233332000
H	-2.300131000	-2.578376000	-1.104227000
O	2.337701000	-2.169978000	0.233370000
H	2.299487000	-2.579028000	1.104151000
O	2.338104000	2.169587000	-0.233312000
H	2.300088000	2.578609000	-1.104115000
O	-2.337737000	2.169906000	0.233328000
H	-2.299622000	2.578842000	1.104166000

Gibbs free energy = -643.867952 Hartree

Number of imaginary frequencies: 0

8a_pl

	angstroms		
	X	Y	Z
N	-0.022250000	-1.779575000	0.032446000
C	-1.280791000	-1.271178000	0.058367000
C	1.770636000	-0.022763000	-0.034109000
C	-1.770643000	0.022771000	0.034871000
C	1.280831000	1.271157000	-0.059605000
N	0.022230000	1.779543000	-0.033068000
H	-0.034551000	-2.792093000	0.070232000
H	0.034487000	2.792014000	-0.072057000

H	-2.854774000	0.036705000	0.056569000
H	2.854797000	-0.036684000	-0.054738000
C	1.249058000	-1.303684000	0.010275000
C	-1.249043000	1.303703000	-0.009042000
O	2.146159000	-2.377667000	0.070844000
H	2.665983000	-2.400048000	-0.739678000
O	2.202400000	2.321594000	-0.155827000
H	2.752698000	2.331407000	0.634588000
O	-2.146352000	2.377675000	-0.067791000
H	-2.664575000	2.400082000	0.743748000
O	-2.202349000	-2.321806000	0.152606000
H	-2.753083000	-2.329546000	-0.637506000

Gibbs free energy = -643.866786 Hartree

Number of imaginary frequencies: 0

8b

	angstroms		
	X	Y	Z
N	1.779916000	-0.000004000	0.000015000
C	1.269555000	-1.242436000	0.000016000
C	0.000004000	1.773900000	0.000035000
C	-0.000003000	-1.773901000	0.000026000
C	-1.269555000	1.242436000	0.000042000
N	-1.779915000	0.000003000	0.000042000
H	2.815227000	-0.000005000	0.000007000
H	-2.815226000	0.000005000	0.000051000

H	-0.000005000	-2.856427000	0.000024000
H	0.000006000	2.856426000	0.000038000
C	1.269560000	1.242432000	0.000025000
C	-1.269559000	-1.242432000	0.000037000
N	-2.390446000	2.243790000	0.000054000
O	-3.533211000	1.791900000	0.000047000
O	-2.106970000	3.426927000	0.000047000
N	2.390455000	2.243781000	0.000017000
O	2.106984000	3.426919000	-0.000120000
O	3.533218000	1.791887000	-0.000145000
N	2.390447000	-2.243789000	0.000006000
O	2.106972000	-3.426927000	0.000083000
O	3.533211000	-1.791898000	0.000077000
N	-2.390456000	-2.243781000	0.000043000
O	-2.106987000	-3.426921000	-0.000148000
O	-3.533219000	-1.791886000	-0.000148000

Gibbs free energy = -1161.184338 Hartree

Number of imaginary frequencies: 0

8c_np

	angstroms		
	X	Y	Z
N	1.777719000	0.000042000	-0.000054000
C	1.298892000	-1.275969000	-0.099928000
C	-0.000033000	1.761020000	-0.000022000
C	0.000042000	-1.761010000	0.000046000

C	-1.298872000	1.275989000	-0.099947000
N	-1.777703000	-0.000033000	0.000000000
H	2.788571000	0.000068000	-0.000114000
H	-2.788553000	-0.000055000	-0.000007000
H	0.000078000	-2.845645000	0.000105000
H	-0.000049000	2.845656000	-0.000031000
C	1.298840000	1.276032000	0.099902000
C	-1.298820000	-1.276029000	0.099941000
C	-2.410616000	-2.272429000	0.367376000
H	-2.730887000	-2.261328000	1.417216000
H	-3.297853000	-2.066502000	-0.246185000
H	-2.090494000	-3.288899000	0.136247000
C	2.410677000	-2.272370000	-0.367381000
H	2.730719000	-2.261491000	-1.417294000
H	3.298049000	-2.066271000	0.245927000
H	2.090656000	-3.288809000	-0.135960000
C	2.410605000	2.272434000	0.367426000
H	2.730560000	2.261576000	1.417366000
H	3.298026000	2.066315000	-0.245805000
H	2.090610000	3.288867000	0.135944000
C	-2.410713000	2.272338000	-0.367374000
H	-2.731059000	2.261151000	-1.417191000
H	-3.297902000	2.066435000	0.246267000
H	-2.090601000	3.288834000	-0.136349000

Gibbs free energy = -500.109299 Hartree

Number of imaginary frequencies: 0

8c_pl

	angstroms		
	X	Y	Z
N	0.007133000	1.784543000	0.000000000
C	1.284841000	1.303305000	0.000000000
C	-1.756578000	0.004666000	0.000000000
C	1.756578000	-0.004666000	0.000000000
C	-1.284841000	-1.303305000	0.000000000
N	-0.007133000	-1.784543000	0.000000000
H	0.007172000	2.793976000	0.000000000
H	-0.007172000	-2.793976000	0.000000000
H	2.841789000	-0.013521000	0.000000000
H	-2.841789000	0.013521000	0.000000000
C	-1.274348000	1.307678000	0.000000000
C	1.274348000	-1.307678000	0.000000000
C	2.320891000	-2.411717000	0.000000000
H	2.966626000	-2.355654000	0.882690000
H	1.869181000	-3.408874000	0.000000000
H	2.966626000	-2.355654000	-0.882690000
C	2.299043000	2.433033000	0.000000000
H	2.197554000	3.074832000	-0.885426000
H	2.197554000	3.074832000	0.885426000
H	3.317253000	2.045736000	0.000000000
C	-2.320891000	2.411717000	0.000000000
H	-2.966626000	2.355654000	0.882690000
H	-1.869181000	3.408874000	0.000000000

H	-2.966626000	2.355654000	-0.882690000
C	-2.299043000	-2.433033000	0.000000000
H	-2.197554000	-3.074832000	-0.885426000
H	-2.197554000	-3.074832000	0.885426000
H	-3.317253000	-2.045736000	0.000000000

Gibbs free energy = -500.106905 Hartree

Number of imaginary frequencies: 2

9a_np

	angstroms		
	X	Y	Z
N	-0.000002000	1.782950000	0.000064000
C	1.265515000	1.287559000	0.059889000
C	-1.782270000	0.005473000	0.015695000
C	1.782275000	0.005477000	-0.015713000
C	-1.263681000	-1.279452000	0.092526000
N	0.000000000	-1.776608000	0.000083000
H	-0.000005000	2.791468000	0.000148000
H	0.000007000	-2.785104000	0.000118000
H	2.013548000	2.064854000	0.127417000
H	-1.999628000	-2.059477000	0.235006000
C	-1.265510000	1.287559000	-0.059827000
C	1.263683000	-1.279447000	-0.092484000
H	1.999600000	-2.059490000	-0.235033000
H	-2.013545000	2.064854000	-0.127343000
O	3.199131000	0.033708000	-0.075428000

H	3.542676000	-0.381901000	0.721402000
O	-3.199146000	0.033651000	0.075255000
H	-3.542583000	-0.381475000	-0.721875000

Gibbs free energy = -493.373723 Hartree

Number of imaginary frequencies: 0

9a_pl

	angstroms		
	X	Y	Z
N	0.001433000	-1.780093000	-0.000675000
C	1.268808000	-1.285388000	0.020558000
C	-1.783552000	-0.001685000	-0.017848000
C	1.783552000	0.001686000	0.017849000
C	-1.268808000	1.285388000	-0.020551000
N	-0.001433000	1.780093000	0.000674000
H	0.002127000	-2.788570000	0.000723000
H	-0.002127000	2.788570000	-0.000724000
H	2.015155000	-2.066399000	0.064583000
H	-2.015156000	2.066400000	-0.064567000
C	-1.266627000	-1.287597000	-0.017233000
C	1.266627000	1.287597000	0.017225000
H	2.012962000	2.068900000	0.051666000
H	-2.012962000	-2.068899000	-0.051684000
O	3.202832000	0.007116000	0.084643000
H	3.545812000	-0.049940000	-0.812599000
O	-3.202832000	-0.007112000	-0.084642000

H	-3.545811000	0.049894000	0.812603000

Gibbs free energy = -493.374775 Hartree

Number of imaginary frequencies: 1

9b

	angstroms		
	X	Y	Z
N	-0.000014000	-1.765944000	0.000029000
C	1.266381000	-1.290084000	0.006947000
C	-1.774625000	0.000008000	0.000001000
C	1.774613000	0.000010000	-0.000001000
C	-1.266404000	1.290104000	0.006927000
N	-0.000013000	1.765962000	-0.000029000
H	-0.000012000	-2.776418000	0.000030000
H	-0.000013000	2.776436000	-0.000028000
H	2.004094000	-2.076070000	0.020704000
H	-2.004126000	2.076085000	0.020675000
C	-1.266407000	-1.290087000	-0.006929000
C	1.266385000	1.290107000	-0.006947000
H	2.004094000	2.076095000	-0.020704000
H	-2.004129000	-2.076068000	-0.020678000
N	3.291565000	-0.000006000	-0.000002000
O	3.872789000	1.068573000	-0.136983000
O	3.872752000	-1.068604000	0.136986000
N	-3.291559000	-0.000005000	0.000001000
O	-3.872750000	1.068585000	0.137058000
O	-3.872716000	-1.068611000	-0.137060000

Gibbs free energy =   -752.039376 Hartree

Number of imaginary frequencies: 0

9c

	angstroms		
	X	Y	Z
N	0.000003000	0.000000000	1.757343000
C	0.000003000	1.275395000	1.275167000
C	-0.002023000	-1.825308000	0.000000000
C	0.002022000	1.825307000	0.000000000
C	-0.000001000	-1.275395000	-1.275167000
N	0.000003000	0.000000000	-1.757343000
H	0.000005000	0.000000000	2.765834000
H	0.000005000	0.000000000	-2.765834000
H	0.005822000	1.985014000	2.092783000
H	-0.005820000	-1.985014000	-2.092783000
C	-0.000001000	-1.275395000	1.275167000
C	0.000003000	1.275395000	-1.275167000
H	0.005822000	1.985014000	-2.092783000
H	-0.005820000	-1.985014000	2.092783000
C	0.069902000	3.356075000	0.000000000
H	-0.424246000	3.778778000	0.878245000
H	1.106580000	3.710350000	0.000000000
H	-0.424246000	3.778778000	-0.878245000
C	-0.069910000	-3.356075000	0.000000000
H	-1.106589000	-3.710345000	0.000000000

H	0.424237000	-3.778779000	-0.878245000
H	0.424237000	-3.778779000	0.878245000

Gibbs free energy = -421.500768 Hartree

Number of imaginary frequencies: 0

9d_np

	angstroms		
	X	Y	Z
N	-0.016750000	-1.763215000	-0.031077000
C	-1.279044000	-1.262284000	0.093239000
C	1.811891000	-0.019182000	-0.002868000
C	-1.811886000	0.019146000	-0.002946000
C	1.279000000	1.262264000	0.093302000
N	0.016752000	1.763198000	-0.031073000
H	-0.026639000	-2.771414000	-0.061554000
H	0.026647000	2.771400000	-0.061529000
H	-1.999961000	-2.057457000	0.235657000
H	1.999966000	2.057430000	0.235545000
C	1.254117000	-1.285852000	-0.126033000
C	-1.254130000	1.285839000	-0.126134000
H	-1.976094000	2.075427000	-0.279458000
H	1.976135000	-2.075456000	-0.279043000
N	-3.266304000	0.094618000	0.009089000
H	-3.693147000	-0.683171000	-0.478982000
H	-3.636195000	0.125889000	0.952722000
N	3.266333000	-0.094672000	0.009199000

H	3.693092000	0.682110000	-0.480568000
H	3.636290000	-0.123845000	0.952876000

Gibbs free energy = -453.603436 Hartree

Number of imaginary frequencies: 0

9d_pl

	angstroms		
	X	Y	Z
N	0.037613000	-1.763207000	0.013519000
C	1.301184000	-1.250277000	0.009419000
C	-1.814961000	-0.042394000	0.000370000
C	1.814961000	0.042394000	-0.000370000
C	-1.301184000	1.250277000	-0.009419000
N	-0.037613000	1.763207000	-0.013519000
H	0.058560000	-2.771570000	0.021170000
H	-0.058560000	2.771570000	-0.021170000
H	2.040443000	-2.041107000	0.015523000
H	-2.040443000	2.041107000	-0.015523000
C	-1.244523000	-1.308509000	0.009987000
C	1.244523000	1.308509000	-0.009987000
H	1.963442000	2.114942000	-0.016174000
H	-1.963442000	-2.114942000	0.016174000
N	3.274440000	0.135560000	-0.001157000
H	3.674842000	-0.298857000	0.822933000
H	3.674786000	-0.311586000	-0.818437000
N	-3.274440000	-0.135560000	0.001157000

H	-3.674786000	0.311586000	0.818437000
H	-3.674842000	0.298857000	-0.822933000

Gibbs free energy = -453.602289 Hartree

Number of imaginary frequencies: 1

9e

	angstroms		
	X	Y	Z
N	0.000018000	-1.761699000	-0.000164000
C	-1.273035000	-1.280143000	0.024269000
C	1.817649000	-0.000005000	0.000001000
C	-1.817647000	-0.000013000	-0.000003000
C	1.273083000	1.280131000	0.024554000
N	0.000010000	1.761681000	0.000165000
H	0.000019000	-2.770602000	-0.000290000
H	0.000008000	2.770583000	0.000295000
H	-1.987898000	-2.089839000	0.077887000
H	1.987952000	2.089810000	0.078329000
C	1.273088000	-1.280144000	-0.024570000
C	-1.273041000	1.280120000	-0.024254000
H	-1.987909000	2.089813000	-0.077854000
H	1.987961000	-2.089819000	-0.078356000
C	-3.323381000	-0.000012000	0.000003000
C	-4.043104000	-0.675128000	-0.994876000
C	-4.043097000	0.675130000	0.994877000
C	-5.436869000	-0.678892000	-0.994142000

H	-3.500605000	-1.185842000	-1.782893000
C	-5.436857000	0.678917000	0.994141000
H	-3.500584000	1.185841000	1.782887000
C	-6.140146000	0.000014000	-0.000001000
H	-5.973496000	-1.202735000	-1.777804000
H	-5.973482000	1.202771000	1.777797000
H	-7.224378000	0.000027000	0.000001000
C	3.323388000	-0.000002000	-0.000002000
C	4.043077000	-0.675733000	0.994477000
C	4.043076000	0.675740000	-0.994481000
C	5.436844000	-0.679513000	0.993730000
H	3.500530000	-1.186932000	1.782150000
C	5.436838000	0.679534000	-0.993728000
H	3.500521000	1.186931000	-1.782152000
C	6.140110000	0.000012000	0.000002000
H	5.973484000	-1.203824000	1.777069000
H	5.973480000	1.203849000	-1.777063000
H	7.224342000	0.000020000	0.000001000

Gibbs free energy = -804.987782 Hartree

Number of imaginary frequencies: 0

10a

	angstroms		
	X	Y	Z
N	1.869210000	0.000026000	0.046677000
C	1.306547000	-1.241270000	0.029374000

C	-0.000037000	1.707511000	0.000022000
C	0.000036000	-1.707551000	0.000022000
C	-1.306547000	1.241230000	-0.029103000
N	-1.869209000	-0.000074000	-0.046694000
H	2.056110000	-2.019903000	0.039615000
H	0.000068000	-2.793997000	0.000034000
H	-2.056135000	2.019848000	-0.039009000
H	-0.000069000	2.793958000	0.000037000
C	1.306502000	1.241313000	0.029110000
C	-1.306501000	-1.241353000	-0.029352000
H	-2.056014000	-2.020026000	-0.039602000
H	2.056040000	2.019971000	0.039010000
C	3.343327000	0.000050000	-0.036387000
H	3.740094000	-0.882760000	0.462896000
H	3.667412000	-0.000072000	-1.080705000
H	3.740059000	0.882988000	0.462694000
C	-3.343328000	0.000072000	0.036345000
H	-3.667429000	0.002056000	1.080656000
H	-3.740070000	0.882001000	-0.464540000
H	-3.740066000	-0.883742000	-0.461149000

Gibbs free energy = -421.483460 Hartree

Number of imaginary frequencies: 0

10b

	angstroms		
	X	Y	Z
N	-1.858594000	0.000005000	0.000000000
C	-1.304546000	1.249295000	-0.027535000
C	0.000005000	-1.718473000	0.001204000
C	0.000012000	1.718477000	-0.001213000
C	1.304567000	-1.249329000	-0.025790000
N	1.858592000	-0.000001000	-0.000004000
H	-2.065353000	2.014066000	-0.083691000
H	0.000012000	2.804823000	-0.001998000
H	2.065391000	-2.014170000	-0.080787000
H	0.000002000	-2.804819000	0.001985000
C	-1.304550000	-1.249285000	0.027532000
C	1.304572000	1.249328000	0.025780000
H	2.065399000	2.014166000	0.080772000
H	-2.065362000	-2.014052000	0.083688000
C	-3.309137000	0.000004000	0.000001000
C	-3.998637000	-0.465484000	-1.116980000
C	-3.998638000	0.465483000	1.116984000
C	-5.392532000	-0.466362000	-1.113308000
H	-3.439548000	-0.816026000	-1.976272000
C	-5.392535000	0.466356000	1.113312000
H	-3.439552000	0.816026000	1.976277000
C	-6.090916000	-0.000003000	0.000002000
H	-5.931268000	-0.823960000	-1.983353000
H	-5.931270000	0.823951000	1.983358000

H	-7.174901000	-0.000006000	0.000001000
C	3.309142000	-0.000002000	-0.000003000
C	3.998622000	-0.463289000	1.117899000
C	3.998628000	0.463287000	-1.117896000
C	5.392519000	-0.464169000	1.114236000
H	3.439516000	-0.812162000	1.977859000
C	5.392527000	0.464165000	-1.114226000
H	3.439530000	0.812163000	-1.977861000
C	6.090899000	-0.000003000	0.000006000
H	5.931258000	-0.820062000	1.984977000
H	5.931268000	0.820059000	-1.984965000
H	7.174885000	-0.000005000	0.000010000

Gibbs free energy = -804.971162 Hartree

Number of imaginary frequencies: 0

10c

	angstroms		
	X	Y	Z
N	1.842042000	-0.231148000	-0.000012000
C	1.457011000	1.079262000	-0.000122000
C	-0.222350000	-1.705671000	0.000293000
C	0.222349000	1.705672000	0.000075000
C	-1.457016000	-1.079237000	0.000154000
N	-1.842028000	0.231152000	0.000187000
H	2.316646000	1.731854000	-0.000384000
H	0.359462000	2.783248000	0.000116000

H	-2.316637000	-1.731852000	-0.000012000
H	-0.359479000	-2.783244000	0.000397000
C	1.125868000	-1.407525000	0.000151000
C	-1.125880000	1.407521000	0.000496000
H	-1.778736000	2.268579000	0.000777000
H	1.778728000	-2.268577000	0.000227000
C	3.267796000	-0.445245000	-0.000112000
O	4.089682000	0.423558000	-0.000318000
H	3.514092000	-1.515985000	0.000076000
C	-3.267823000	0.445246000	-0.000122000
O	-4.089659000	-0.423580000	-0.000605000
H	-3.514093000	1.515995000	0.000091000

Gibbs free energy = -569.605698 Hartree

Number of imaginary frequencies: 0

10d

	angstroms		
	X	Y	Z
N	1.865678000	0.056472000	-0.000621000
C	1.304156000	-1.196915000	0.000095000
C	-0.000020000	1.758746000	-0.000247000
C	0.000043000	-1.653067000	-0.000284000
C	-1.308557000	1.308753000	0.000816000
N	-1.865661000	0.056386000	0.000318000
H	2.052686000	-1.973148000	0.000867000

H	0.000064000	-2.738863000	-0.000395000
H	-2.047205000	2.089651000	0.001965000
H	-0.000041000	2.844512000	-0.000335000
C	1.308528000	1.308820000	-0.001202000
C	-1.304103000	-1.196973000	-0.000567000
H	-2.052576000	-1.973251000	-0.001363000
H	2.047165000	2.089732000	-0.002406000
C	3.322990000	-0.013344000	0.000145000
C	-3.323008000	-0.013324000	0.000098000
F	3.903629000	1.188015000	-0.002002000
F	3.767303000	-0.673735000	1.084694000
F	3.768250000	-0.677912000	-1.081473000
F	-3.903534000	1.188018000	0.001795000
F	-3.767963000	-0.677366000	1.082188000
F	-3.767728000	-0.674222000	-1.084017000

Gibbs free energy = -1017.173889 Hartree

Number of imaginary frequencies: 0
